# Adaptive optics in the Arctic? A commentary on Fosbury and Jeffery

**DOI:** 10.1098/rspb.2022.1528

**Published:** 2022-09-28

**Authors:** Nathaniel J. Dominy, Julie M. Harris

**Affiliations:** ^1^ Departments of Anthropology and Biological Sciences, Dartmouth College, 6047 Silsby Hall, Hanover, NH 03755-3537, USA; ^2^ Zukunftskolleg, University of Konstanz, Box 216, Konstanz 78457, Germany; ^3^ School of Psychology and Neuroscience, University of St Andrews, South Street, St Andrews, Fife KY16 9JP, UK

## 1. Introduction

Obscure and unheralded in the annals of visual neuroscience, the reindeer (*Rangifer tarandus*) is having a moment thanks to the work of Fosbury & Jeffery [1]. Their paper pulls on several threads at once to unravel the physiology and functional ecology of two ocular oddities. The first is a colour-shifting tapetum lucidum, the retinal tissue responsible for ‘eye shine’. This mirror-like tissue changes from a mammal-typical golden hue during the summer months to a vivid liquescent blue during the winter months, only to reverse its reflecting properties again with the onset of summer [2]. Tapeta enhance visual sensitivity under low light levels and are therefore widespread among nocturnal animals, but only those of reindeer are known to change seasonally, and it was this extraordinary plasticity that motivated Fosbury & Jeffery [1] to investigate the underlying mechanisms.

Another enigma concerns the reindeer's cornea and lens. Overexposure to ultraviolet (UV) light can cause irreversible damage to retinal photoreceptors [3], so most diurnal mammals have UV-filtering ocular media [4]. In reindeer, however, the cornea and lens transmit up to 60% of available UV light [5], which is enough to excite the photoreceptors responsible for vision [5]. The advantages of this trait are uncertain [5,6] as most mammals with UV-sensitive photoreceptors––some rodents, bats and marsupials––are strongly nocturnal [7] with few exceptions [8], and thus avoid retinal damage by minimizing their exposure to intense daylight. But reindeer are day-active ungulates that live at Arctic and subarctic latitudes, habitats that expose their retinae to high levels of UV radiation during the extended photoperiods of summer [9]. Also, snow is another problem––it is the most reflective natural surface on Earth, and peak albedo effects can nearly double the amount of UV light entering reindeer eyes [10].

## 2. Angles in the atmosphere

**Figure 1.**
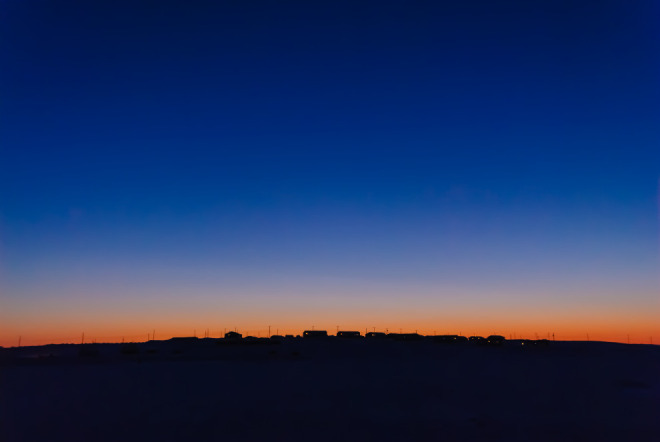
The village of Puvirnituq, Canadian Arctic. At these latitudes, the violet-blue colour of twilight is prolonged, dominating ambient light conditions for 8–11 h each day during the winter months. Photograph by Stephen Gorman, reproduced with permission.

So what to make of these twin puzzles? For starters, Bob Fosbury, an astrophysicist, and Glen Jeffery, a visual neuroscientist, focused on the light environments of circumpolar winters. They described how low solar elevations (between 0° and −18°) increase the path length of sunlight through stratospheric ozone, which selectively attenuates green and yellow wavelengths. In consequence, the dominant colour of twilight is a vivid violet-blue. Termed the blue hour by photographers, gloaming light is a fleeting phenomenon for most organisms, but for those living above 70° latitude, it accounts for 8–11 h of each day between September and April (figure 1). Fosbury and Jeffery described this prolonged period of blue light as ‘extended twilight’, and it motivated them to compare its irradiance spectrum, which peaks around 450 nm, with the photonic properties of reindeer tapeta.

Summer and winter eyes of reindeer were obtained from a Norwegian slaughterhouse and dissected to extract the tapeta, which Fosbury and Jeffery submerged in water to minimize specular distortions, an improvement on previous efforts [2]. The reflectivity of summer tapeta was relatively uniform, with a broad peak (*λ*_max_) of *ca* 650 nm. The *λ*_max_ of winter tapeta varied across the regions of greatest visual acuity; i.e. the centre of the retina (the *area centralis*; *ca* 480 nm) and the horizontal meridian (corresponding to the visual streak; *ca* 450 nm). In all cases, the reflective efficacy of the tapetum extended to 350 nm, the point at which collagen begins to absorb UV light. Thus, winter tapeta are extremely well suited for maximizing UV and blue visual sensitivity under the irradiance of extended twilight.

This striking degree of spectral congruence suggests an adaptive function, namely to optimize visual contrasts of critical objects under twilight conditions [5,6]. But what are the underlying mechanisms at work, and what are the essential selective pressures? Fosbury and Jeffery tackled these questions, too, and with great elegance.

## 3. Packing for winter

Typologists have classified the reindeer's tapetum as a *tapetum fibrosum* because the reflective material is an array of highly ordered, hexagonally packed, collagen fibrils arranged in lamellae of varying thickness [11], and it is the diameters and regular packing structure of these fibrils that determines both the amount and *λ*_max_ of reflected light [12]. Fosbury and Jeffery's insight was to recognize that the packing of these fibrils could be indirectly affected by summer-winter differences in internal eye pressure.

To explore this idea, they developed a physical model of the two-dimensional photonic crystal structure. When intraocular pressures are low during summer months, their model predicts fluid infilling between fibrils and thus wider inter-fibril distances; but when intraocular pressures are high during the winter months, some of the fluid between fibrils is expelled, resulting in a compacted hexagonal array of fibrils. Changes in fibril spacing predict changes in *λ*_max_, which they tested by slowly evaporating fluid from summer and winter tapetum surfaces while monitoring changes in the reflectance spectrum. This experiment confirmed that changes in fibril packing caused shifts in tapetal colour, from the golden-turquoise of summer to the deep blue of winter.

## 4. Visual ecology in the land of long shadows

Fosbury & Jeffery's study [1] of pressure-mediated changes to the *λ*_max_ of reindeer tapeta is certain to find a place in the Hall of Phenotypic Plasticity, and they are to be commended for solving a photonic puzzle. However, is the winter tapetum of reindeer truly ‘tuned,’ as they put it, to the blue colour of extended twilight? Or is it better viewed as a spandrel? (biologists' jargon for a trait that is a byproduct of selection on another trait). The answer pivots in part on the factors that mediate variation in intraocular pressure. One idea is that increasing pressure stems from sustained pupil dilation under the low light conditions, which may block ocular drainage. If true, then the twilight colour-matching of winter tapeta is a spurious coincidence, a side-effect of the autonomic pupillary reflex. By this reasoning, the functional advantages of blue tapeta emerged only after selection operated on the UV transmission properties of the ocular media, at which point it became an exaptation.

**Figure 2.**
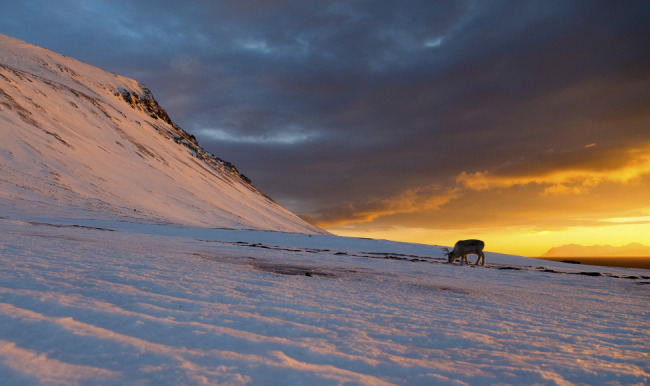
A reindeer forages on terricolous lichens at the onset of twilight in Svalbard, Norway. Reindeer visual systems appear well suited for detecting patches of UV-absorbing lichens on snowy landscapes [6]. Photograph by Espen Bergersen, reproduced with permission.

It is useful, as Fosbury and Jeffery did, to view the winter tapeta and ocular media of reindeer as coupled systems, and to consider the fitness benefits of discriminating vital objects under twilight conditions. Some authors have discussed the value of seeing UV-absorbing foods against a background of UV-reflecting snow (figure 2), but Fosbury and Jeffery focused instead on wolves, the primary predator of reindeer. They measured the spectral reflectance of white hair from huskies as a proxy for wolf hair, and they found that it absorbs UV wavelengths. This result supports the idea that UV-absorbance is a basic property of keratin [13], the hair-fibre protein, and it suggests that reindeer can readily discriminate wolves from snow using UV contrasts. The hair of polar bears, for example, is a strong absorber of UV light, in contrast to snow, which is why UV photography shows polar bears as black against a white snow background [14].

Did better predator detection exert selective pressure on the UV-sensitivity of reindeer visual systems? It is a compelling hypothesis, but it raises a host of questions given that several other ungulates––moose (*Alces alces*), muskoxen (*Ovibos moschatus*), roe deer (*Capreolus capreolus*)––are susceptible to the depredations of wolves in circumpolar regions. What are the optical properties of their ocular media, and do their tapeta change colour seasonally? Investigating the visual systems of these species, along with those of two icons of concealing coloration, Arctic hares (*Lepus arcticus*) and foxes (*Vulpes lagopus*), may prove rewarding. If UV-transmitting eyes are widespread among Arctic mammals, then the benefits must outweigh the costs of UV-damage to the eye. Yet, ‘snow blindness’ is unknown among reindeer, which hints at photoprotective mechanisms in the eye, such as the upregulation of ascorbic acid [15]. This topic invites further study.

The evolution and functional ecology of phenotypic plasticity is a highly debated issue in evolutionary biology, and one wonders if Fosbury and Jeffery's study is destined to become a textbook example of plasticity in response to environmental conditions. However on balance, their work provokes more questions than it answers, and we thank them for it. Their findings are a testament to the benefits of creative cross-disciplinary collaboration, and they will almost certainly stimulate further research and discovery on the evolution and visual ecology of Arctic organisms. Who knows, maybe the extraordinary photonics of reindeer tapeta will inspire the design of solar arrays in circumpolar regions.

## Data Availability

This article does not contain any additional data.
